# Feasibility of a Short Message Service based intervention to improve adherence among HIV-positive youth in Uganda: a focus group study

**DOI:** 10.1186/1471-2334-14-S3-E45

**Published:** 2014-05-27

**Authors:** Yashodhara Rana, Barbara Mukasa, Andrew Kambugu, Peter Wabukala, Crystal Huang, Glenn J Wagner, Sebastian Linnemayr

**Affiliations:** 1 RAND Corporation, Santa Monica, CA, USA; 2 MILDMAY Uganda, Kampala, Uganda; 3 Infectious Diseases Institute, Kampala, Uganda

## Background

More than half of those newly infected with HIV are young people yet youths have lower medication adherence than adults. Short message service (SMS) based interventions have demonstrated their effectiveness in improving adherence among adults in resource limited settings, but their efficacy with youths has not been studied. This study is one of the first qualitative studies to explore youths view on strategies for best implementing SMS based interventions.

## Methods

Six focus groups were conducted with 20 male and 19 female HIV-positive youths in Kampala, Uganda. Participants discussed barriers to adherence, availability and acceptability of SMS messages and strategies for implementation. Verbatim transcripts were managed in ATLAS.ti and analyzed using a framework approach.

## Results

We found that major barriers to adherence were forgetfulness, stigma, poor social support, inadequate food supply and stressful working conditions. Participants reasoned that SMS based interventions would improve adherence by providing them with much needed reminders and social support. However, youths noted that the success of such interventions hinged on: i) ensuring HIV status anonymity given that text messages are vulnerable to security threats ii) developing effective content and having appropriate delivery times so that youth remain engaged iii) increasing accessibility to include youth without phones iv) addressing challenges related to phone sharing and restrictions on the use of phones by younger adolescents.

## Conclusion

SMS based interventions have the potential to promote adherence among youths. The primary recommendation of this study is to ensure confidentiality and increase accessibility.

